# Melatonin augments anti-tumor activity and alleviates nephrotoxicity of gemcitabine in a pancreatic cancer xenograft model targeting P62/Keap1 pathway

**DOI:** 10.1007/s00210-025-03938-x

**Published:** 2025-03-18

**Authors:** Samar Ibrahim, Eman H. Yousef, Ahmed M. El-Dessouki, Nahed A Raslan, Amany A. Alzokaky

**Affiliations:** 1 Pharmacy Practice and Clinical Pharmacy Department, Faculty of Pharmacy, Galala University, Ataka, Egypt; 2 Pharmacology and Biochemistry Department, Faculty of Pharmacy, Horus University-Egypt, New Damietta, 34518 Egypt; 3 Pharmacology and Toxicology Department, Faculty of Pharmacy, Ahram Canadian University, Giza, 12566 Egypt; 4 Pharmacology and Toxicology Department, Faculty of Pharmacy (Girls), Al-Azhar University, Cairo, 11651 Egypt; 5 Department of Clinical Pharmacy Program, College of Health Sciences and Nursing, Al-Rayan Colleges, AL-Madina AL-Munawarah, Saudi Arabia

**Keywords:** Gemcitabine, Melatonin, Pancreatic cancer, Nephrotoxicity, Oxidative stress, Autophagy, Keap1/p62 pathway

## Abstract

Although gemcitabine is a primary chemotherapy for pancreatic cancer, its effectiveness is limited by chemoresistance and nephrotoxicity, posing significant clinical challenges. Therefore, the development of novel therapeutic approaches to prevent pancreatic malignancy remains crucial. This study aimed to investigate the potential of melatonin in enhancing gemcitabine’s anticancer efficacy while mitigating its nephrotoxic effects through modulation of the Keap1/p62 pathway. A pancreatic cancer xenograft model was established in rats, which received either gemcitabine (50 mg/kg, I.P.), melatonin (50 mg/kg, I.P.), or their combination three times per week for 2 weeks. Our findings demonstrate that melatonin potentiates gemcitabine’s cancer-suppressing effects via modulation of the Kelch-like-ECH associated protein-1 (Keap1)/p62 pathway, resulting in reduced fibrosis, oxidative stress, and inflammatory markers. Additionally, melatonin significantly mitigated gemcitabine-induced nephrotoxicity. These results suggest that melatonin may serve as an adjuvant therapy in pancreatic cancer treatment, enhancing chemotherapy efficacy while reducing its adverse effects.

## Introduction

In recent decades, the advancement in cancer treatments has significantly enhanced survival rates (Siegel et al. 2018). However, despite these advancements, numerous patients continue to experience recurrence and distal metastasis that remain resistant to treatment, thereby severely diminishing overall survival (Shan et al. 2020). Specifically, pancreatic cancer (PC) stands out as a highly aggressive malignancy, characterized by a substantial likelihood of metastasis, recurrence, and poor prognosis (Fang et al. 2018). Currently, it ranks as the fourth most common cause of cancer-related deaths worldwide (Li et al. 2024). Due to the absence of clear symptoms or the presence of nonspecific signs, the majority of PC cases is diagnosed at an advanced stage, often when surgical intervention is no longer viable. As a result, chemotherapy has become a crucial and indispensable approach in the management of PC (Wood et al. 2022).

Gemcitabine (Gem), a pyrimidine antimetabolite, is among the limited chemotherapy agents demonstrated to enhance the therapeutic efficacy of PC. It has been shown to alleviate clinical symptoms, enhance patients’ quality of life, and extend survival to some degree (Beutel and Halbrook 2023). The primary mechanism through which Gem exerts its anti-tumor effects is by inducing DNA damage; however, tumor cells’ ability to enhance DNA repair often leads to resistance to Gem (Chakraborty et al. 2018, Yang et al. 2021). Additionally, reduced cellular uptake and increased drug efflux are critical factors contributing to this chemoresistance (Bai et al. 2016). The growing challenges of chemoresistance and nephrotoxicity during Gem treatment in clinical settings reduce its effectiveness in treating PC (Li et al. 2020, Beutel and Halbrook 2023). Consequently, it is of ultimate importance to investigate the unrevealed molecular mechanisms underlying PC and to identify safe and effective targeted therapies for its clinical management.

The stress-inducible adaptor protein p62, also referred to as sequestosome-1 (SQSTM1), is a multifunctional ubiquitin-binding protein that participates in numerous cellular processes, such as autophagy, cell growth, oxidative stress response, and mitosis (Uysal et al. 2023, Armeli et al. 2024). Acting as an adaptor in the autophagy pathway, p62 facilitates the connection between ubiquitinated proteins and the autophagic machinery, promoting their degradation. p62 is integral to signaling networks that regulate cell proliferation and survival (Shin et al. 2020, Wang et al. 2021). Abnormal expression of p62 has been linked to the onset and advancement of several cancers, including lung, endometrial (Zhu et al. 2018, Tang et al. 2021), neck and head squamous cell carcinoma (Noman et al. 2020, Wang et al. 2020), and prostate cancer (Jiang et al. 2020). Increased levels of p62 are frequently associated with poor prognosis in cancers like hepatocellular carcinoma, breast cancer, and epithelial ovarian cancer (He et al. 2018, Wang et al. 2018, Liu et al. 2023). Furthermore, p62 has been recognized as a prognostic marker in melanoma (Karras et al. 2019) and is also associated with the development of neurodegenerative diseases, including Alzheimer’s and Parkinson’s (Dong et al. 2022). In addition to its role in autophagy, p62 also functions in a manner independent of autophagy, playing a significant part in tumorigenesis, cancer progression, and chemotherapy resistance (Tang et al. 2021, Zhang and Costa 2021).

Melatonin (N-acetyl-5-methoxytryptamine, Mel) is a hormone produced by the pineal gland that plays a crucial role in controlling various essential physiological functions, such as body temperature, seasonal reproduction, immune response, and circadian rhythms (Sarisozen et al. 2023, Afzal 2024). Numerous studies have highlighted the protective effects of Mel in models of oxidative stress, attributed to both its direct scavenging of free radicals and its indirect antioxidant properties (Monteiro et al. 2024). Additionally, Mel has been found to exert anti-tumor effects in several cancers, including breast, prostate, and lung cancers (Reiter et al. 2024). Recent research suggests that Mel enhances the anti-tumor efficacy of Gem in PC by modulating apoptotic pathways (Leja-Szpak et al. 2018) and inhibiting nuclear factor-κB (NF-κB) activation (Ju et al. 2016). However, the underlying mechanisms remain inadequately elucidated. Moreover, the protective effects of Mel against Gem-induced nephrotoxicity remain unexplored. Consequently, this study aimed to investigate the potential of melatonin in enhancing gemcitabine’s anticancer efficacy while mitigating its nephrotoxic effects through modulation of the Keap1/p62 pathway.

## Materials and methods

### Docking of melatonin to p62

The crystal structure of p62 (PDB ID: 6MJ7) was sourced from the Protein Data Bank (https://www.rcsb.org/pdb/home/home.do). Prior to analysis, the protein structure was preprocessed by removing water molecules and associated complexes. Additionally, hydrogen atoms were added to refine the ionization states and tautomeric forms of amino acid residues. The binding site was determined by identifying a region containing an arginine residue. The three-dimensional configuration of melatonin was accessed via the ZINC database, where its chemical structure was refined by optimizing bond orders, assigning appropriate charges, and adding hydrogen atoms.

Energy minimization was conducted through a conformational search or geometry clean-up process. Before adding hydrogen atoms, adjustments were applied to bond orders and charges. To enhance readability within the AutoDock software, the protein and ligand structures were transformed into PDBQT format. The molecular docking process was carried out at the arginine binding site using AutoDock Vina, with grid map coordinates centered at 0.317407, 4.244889, and 3.235481 along the X, Y, and Z axes, respectively. The binding modes and interactions within the binding pocket were analyzed and visualized employing Chimera version 1.15 and Discovery Studio Visualizer v21.1.0.20298.

### Drugs, chemicals, antibodies, and cell culture

Melatonin (Mel) was sourced from Sigma-Aldrich, Inc. (St. Louis, MO, USA), while Gemcitabine (Gemzar) was provided by Eli Lilly. Antibodies specific to p62 and Kelch-like-ECH associated protein-1 (Keap1) were purchased from Cell Signaling Technology Inc. (Beverly, MA, USA). Pancreatic cancer cell line (PANC-1) was cultured under standard conditions at the National Cancer Institute in Cairo, Egypt. For injection purposes, the cells were prepared in sterile Phosphate-Buffered Saline (PBS) at concentrations ranging between 5 × 10^6^ to 1 × 10^7^ cells per 100–200 μL.

### Animals, xenograft rat model, treatment, and biological sample collection

Thirty male albino Wistar rats, each weighing 200 ± 20 g, were obtained from the animal house at VACSERA, located in Dokki, Giza, Egypt. The animals were housed under standard laboratory conditions, which included a 12-h light/dark cycle, 65% ± 5% relative humidity, and a controlled temperature of 25 °C ± 2 °C. All animal experiments were conducted following approval from the Research Ethics Committee of the Faculty of Pharmacy, Horus University, Egypt (Ethics code: A5/2024). The study adhered to the “Principles of Laboratory Animal Care” (NIH Publication No. 85-23, revised 1985) to ensure ethical treatment and proper care of the animals.

**Fig. 1:**
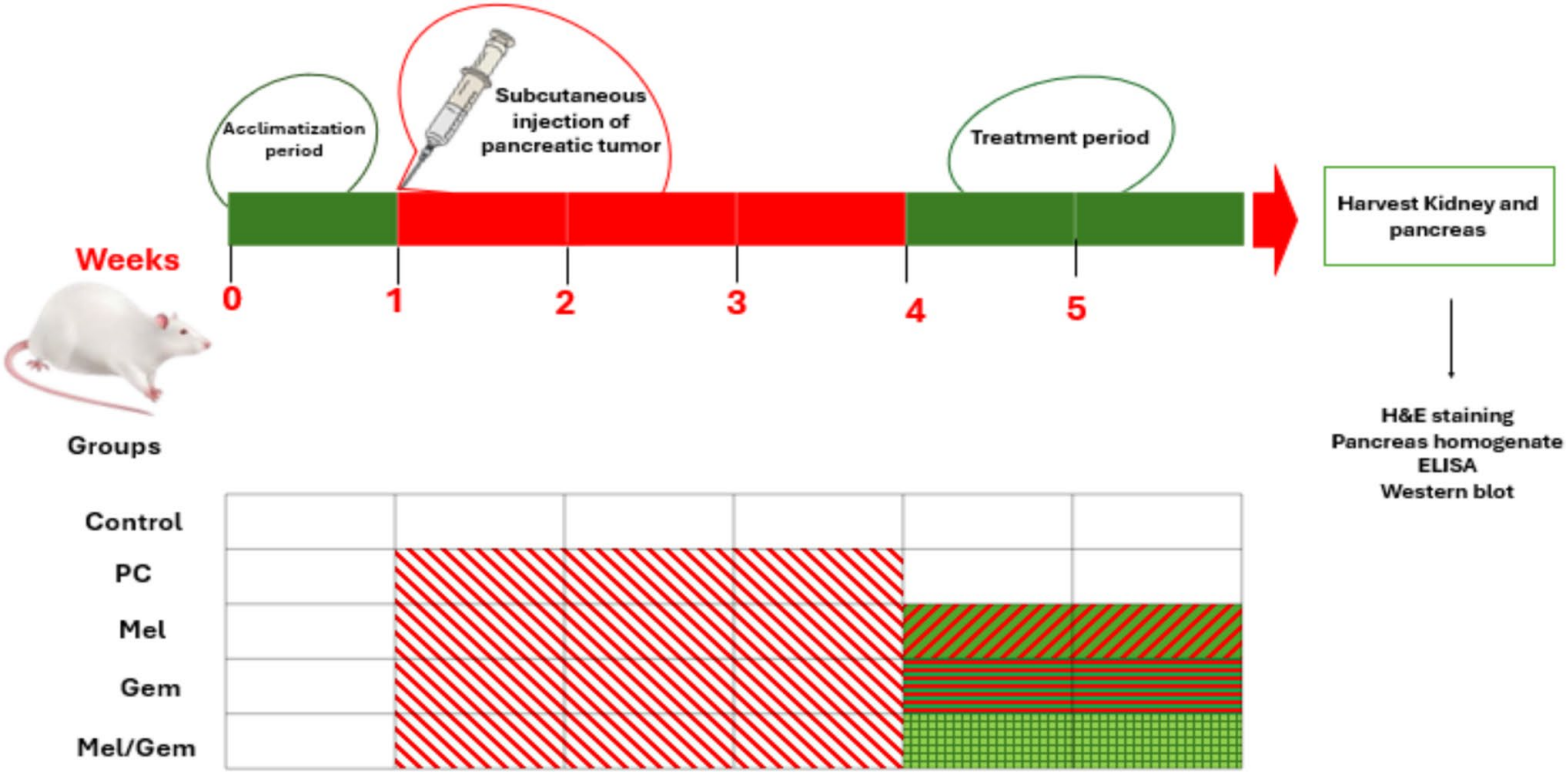
Experimental design. A rat pancreatic cancer xenograft model was established through subcutaneous injection of 100 μL of a tumor cell suspension (5 × 10^6^ to 1 × 10^7^ cells) into the right flank. Three weeks after injection, the rats were treated for two weeks with either melatonin (50 mg/kg, i.p., administered three times weekly), gemcitabine (50 mg/kg, i.p., three times weekly), or a combination of both agents. Following the treatment period, pancreatic and kidney tissues were harvested for further experimental analysis

A tumor xenograft model was developed by subcutaneously injecting 100 μL of the prepared cell suspension into the right flank of each rat. Three weeks after injection, the animals were randomly assigned to five groups, each consisting of six rats. The treatment regimens included a vehicle control (intraperitoneal (i.p.) injection of physiological saline), Mel, Gem, or a combination of Mel and Gem, as outlined in Fig. 1. The administered doses were Mel (50 mg/kg i.p., three times per week for 2 weeks) (Kandemir et al. 2017, Tung et al. 2020) and Gem (50 mg/kg i.p., three times per week for 2 weeks) (Chen et al. 2018). Prior to sacrifice, the rats were anesthetized with pentobarbital sodium (40 mg/kg, i.p.). Following euthanasia, kidneys and pancreatic tumors were carefully excised, washed with ice-cold phosphate-buffered saline (PBS), and divided into three parts. One segment of the pancreas was preserved in 10% buffered formalin for histopathological analysis. The second segment was homogenized at 10% (wt./vol) in ice-cold 0.01 M PBS (pH 7.4) using an Omni-125 handheld homogenizer (Omni International, GA, USA). The homogenate was centrifuged, and the resulting supernatant was stored at − 80 °C for subsequent analyses of oxidative stress markers and enzyme-linked immunosorbent assay** (**ELISA) assays. Kidney tissues were similarly fixed in 10% buffered formalin for histopathological evaluation.

### Histopathological analysis

Pancreatic and kidney tissues were immediately preserved in 10% neutral buffered formalin for 24 h post-collection. Following fixation, the samples underwent washing, sequential dehydration through a graded ethanol series, xylene clearing, and paraffin embedding. Tissue sections, each 5 μm thick, were prepared using a microtome, mounted on glass slides, and dewaxed. To ensure unbiased analysis, the slides were coded anonymously for blinded assessment. The sections were stained with hematoxylin and eosin (H&E) and examined under a light microscope. Histopathological changes in the treatment groups were recorded and imaged using a computer-assisted digital imaging system integrated with a Nikon Digital Camera (Japan).

### Oxidative stress assessment

Oxidative stress in pancreatic tissue homogenates was assessed by measuring the levels of the following:

Malondialdehyde (MDA) (Cat. #: MBS268427, San Diego, USA), by the estimation of thiobarbituric acid reactive substances (TBARS) from the reaction of thiobarbituric acid in acidic medium with TBARS to develop pink color determined at two wavelengths 520 and 535 nm, by the method of Mihara and Uchiyama (1978).

Catalase (CAT) (Cat No. MBS2600683) was evaluated by using 20 μL of the samples and was mixed with 20 μL of catalase followed by addition of 20 μL of dilute hydrogen peroxide to start the reaction. After 20-min incubation, the reaction was terminated by adding 30 μL of diluted potassium hydroxide at room temperature for 10 min. Ten microliters of catalase potassium periodate was added to the samples and left for another 5-min incubation, and the absorbance was read at 540 nm, by the method of Wheeler et al. (1990).

Superoxide dismutase (SOD) (Cat No. CSB-E08555r) was evaluated by using 10 μL of the sample and was mixed with 200 μL of diluted radical detector, the reaction was started by adding 20 μL of diluted xanthine oxidase, and after 20 min of incubation, the absorbance was read at 440–460 nm, by the method of Liu (1996).

Reduced glutathione (GSH) (Cat. #: CEA294Ge, Katy, USA) was determined using Ellman’s reagent [5,5′-dithiobis (2-nitrobenzoic acid)] (DTNB) to develop a stable yellow product measured at 412 nm colorimetrically, by the method of Beutler, Duron (Beutler et al. 1963).

### Enzyme-linked immunosorbent assay (ELISA) analysis

Pancreatic protein levels of transforming growth factor beta-1 (TGF-β1), tumor necrosis factor alpha (TNF-α), interleukin-1β (IL-1β), and interleukin-10 (IL-10) were measured in the supernatant of tissue homogenates using enzyme-linked immunosorbent assay (ELISA) kits, following the protocols provided by the manufacturer (Life Span Bio Sciences, USA).

### Western blot analysis

Protein expression levels of p62 and Keap1 in pancreatic tissue samples were assessed. The rat tissue or cell lysate mixture, consisting of RIPA buffer (Thermo Fisher Scientific, 89900, USA), protease inhibitor (Sigma-Aldrich, P8340, USA), and phosphatase inhibitor (Sigma-Aldrich, P8340, USA), was homogenized to extract total protein. The BCA kit (Thermo Fisher Scientific, 23225, USA) quantified the protein samples before boiling them in Laemmli buffer (Bio-Rad, 1610747, USA) at 95 °C for 5 min. Sodium dodecyl sulfate–polyacrylamide gel electrophoresis (SDS-PAGE) was used to separate proteins from different groups and markers (Bio-Rad, 4561094, USA). The proteins were then transferred to PVDF membranes (Millipore, IPVH00010, USA). Protein concentrations were measured by the Bradford method. Equivalent amounts of cell lysates were fractioned on 8% SDS-PAGE gel and then transferred to nitrocellulose membranes (Bio-Rad). The membranes were blocked in 5% non-fat dried milk in TBST (0.05% Tween-20 Tris-buffered saline) for 1 h and 30 min, followed by incubation with primary antibodies against p62 and Keap1 (dilution 1:200) and β-actin (dilution 1:5000), at 4 °C overnight. Detection of antigen-antibody complexes was achieved using a horseradish peroxidase-conjugated secondary antibody (1:5000, Bio-Rad) for 1 h at room temperature, followed by additional washing, and the chemiluminescence signals were captured using a CCD camera-based imaging system. The expression levels of the proteins were expressed relative to β-actin, serving as a control, and quantified using the ChemiDoc MP imaging system.

### Statistical analysis

Statistical analysis was conducted using one-way ANOVA followed by Tukey’s post hoc test. Data analysis and visualization were performed using GraphPad Prism version 6.01 (GraphPad Software, San Diego, CA, USA). Results are expressed as the mean ± standard error of the mean (SEM), with a *P*-value ≤ 0.05 considered statistically significant.

## Results

### Melatonin molecular interaction study

**Fig. 2:**
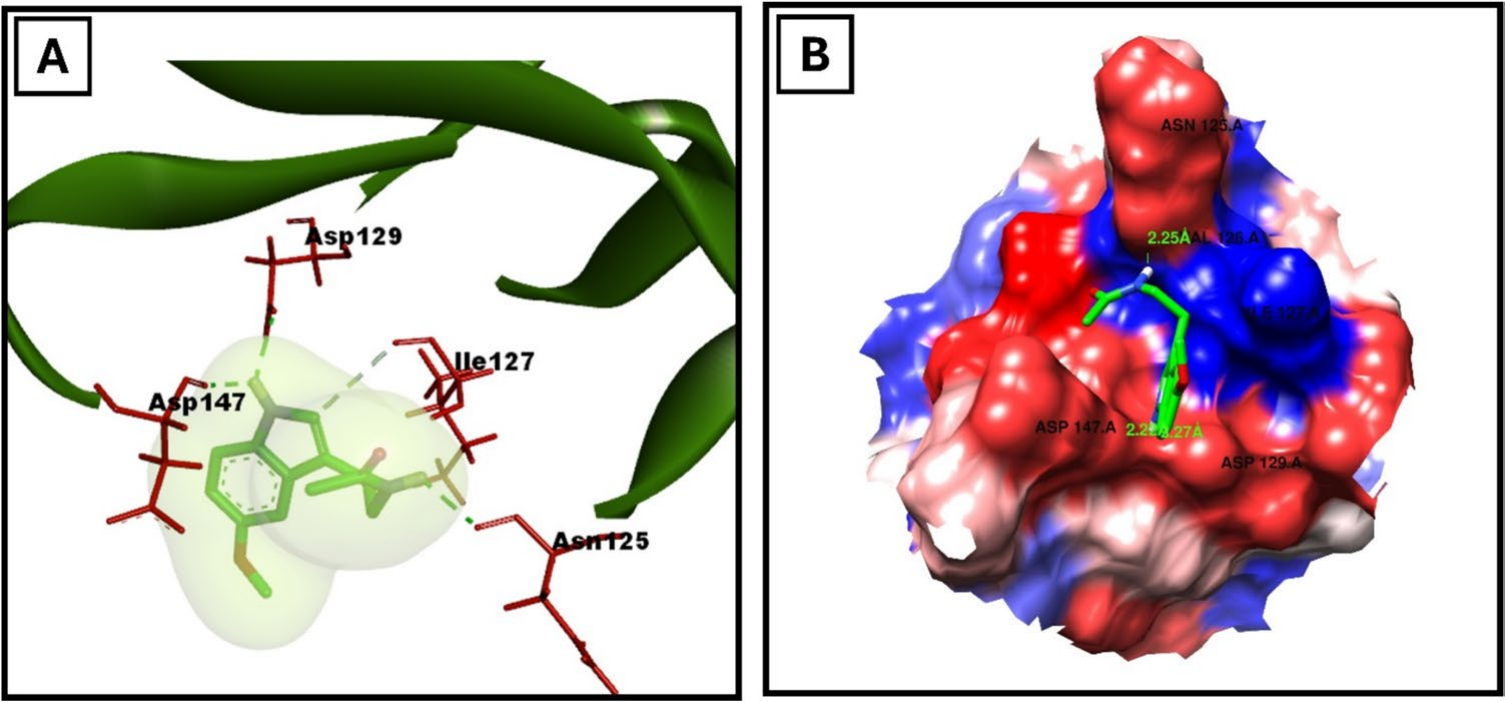
Docked pose of melatonin in the binding pocket of p62. **A** The amino acids participating in the interaction between p62 (PDB ID: 6MJ7) and melatonin were identified and visualized using Discovery Studio Visualizer. **B** Hydrophobic interactions within the p62 binding pocket and melatonin were analyzed and illustrated using Chimera

The binding interactions between melatonin and p62 were assessed using AutoDock Vina 1.1.2 (Trott and Olson 2010). The binding affinity of melatonin to p62 at the arginine binding site was calculated to be − 4 kcal/mol. Three hydrogen bonds were formed between melatonin and the residues ASN125, ASP129, and ASP147. Additionally, the binding of melatonin to p62 was facilitated by hydrophobic interactions with VAL126 and ILE127. The docking results are summarized in Fig. 2 and Table 1.

**Table 1:**
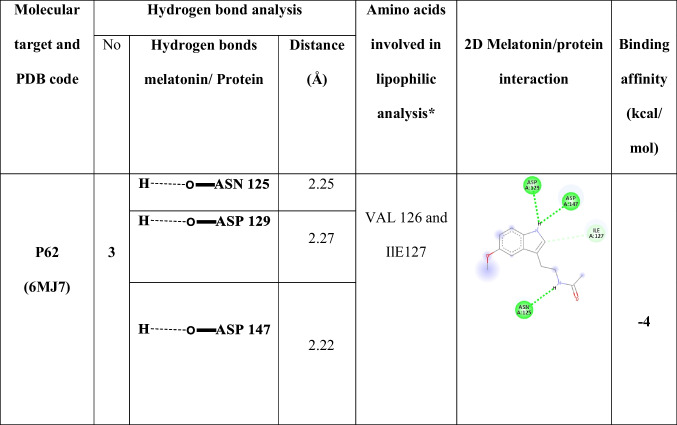
The binding energy, interaction type, and amino acids involved in interaction of p62 with melatonin

### Melatonin enhances Gem anti-tumor activity

**Fig. 3:**
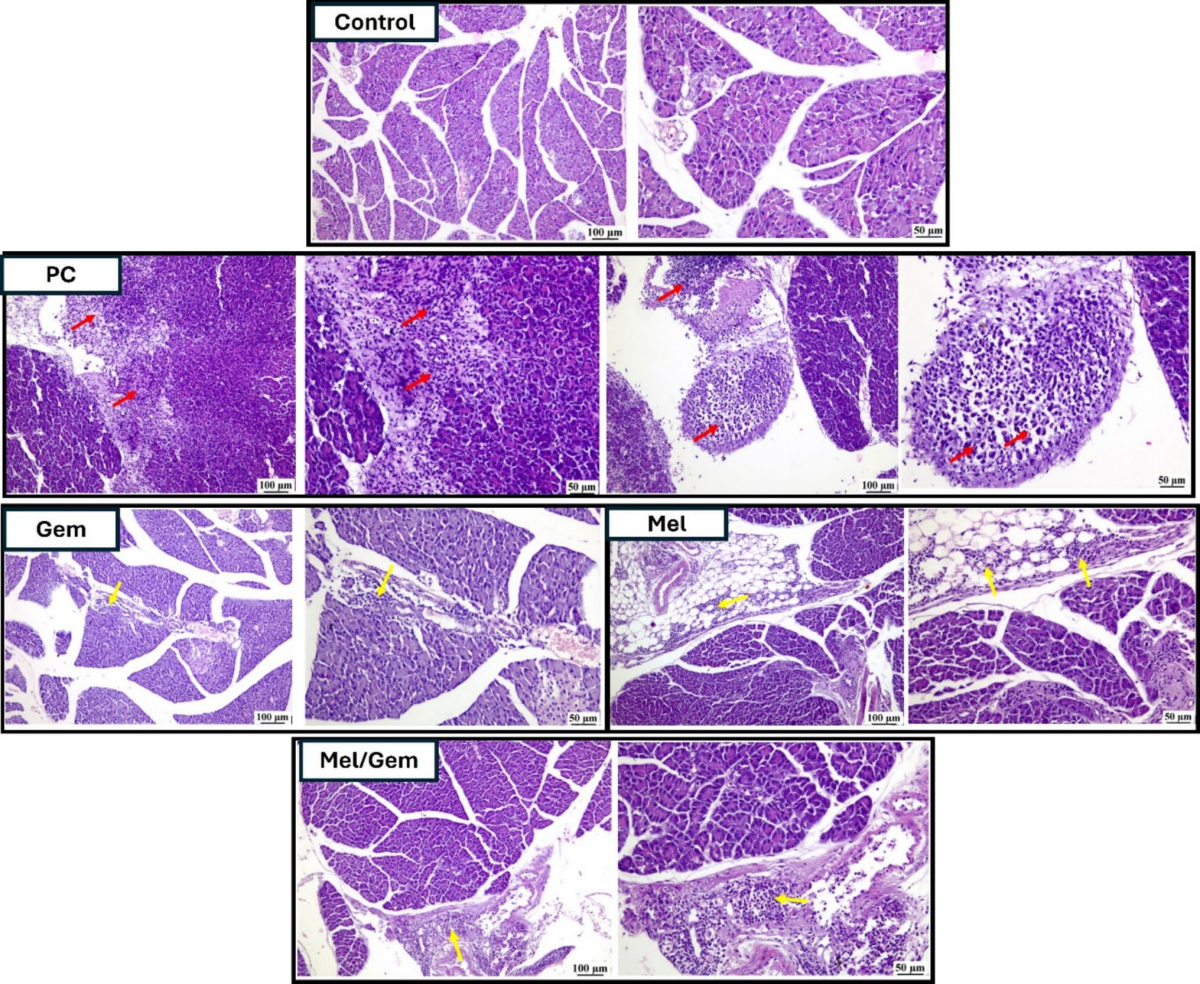
Melatonin was found to enhance the anti-tumor efficacy of Gem. The impact of Mel on pancreatic cancer progression was evaluated across different groups through histopathological analysis of H&E-stained pancreatic tissue. Black arrows in the images indicate fibrous septa, red arrows highlight neoplastic cell populations infiltrating pancreatic tissues, and yellow arrows denote areas of mononuclear inflammatory cell infiltration

Histological examination of H&E-stained pancreatic sections (Fig. 3) from the control group demonstrated a typical pancreatic structure, including normal endocrine and exocrine acini. Contrariwise, sections from the PC group displayed significant pathological alterations, including a proliferation of neoplastic cells infiltrating the pancreatic islets. These cells exhibited distinct malignancy features such as pleomorphism, nuclear hyperchromasia, and frequent atypical mitotic figures. Additionally, the pancreatic acini showed evidence of degeneration and necrosis, accompanied by prominent infiltration of inflammatory cells. In the Gem-treated group, substantial improvement was observed, as sections showed mild infiltration of periductal mononuclear inflammatory cells, with many regions appearing histologically normal. The Mel-treated group demonstrated moderate improvement, characterized by pronounced mononuclear inflammatory cell infiltration in the peripancreatic fat, extending to the periductal regions, along with evident acinar degeneration and necrosis. Notably, the Mel/Gem combination group exhibited remarkable recovery, where the inflammatory response was primarily localized to the peripancreatic tissues, and the pancreatic islets and acini appeared largely normal.

### Melatonin/Gem modulates p62/keap1 signaling pathway

**Fig. 4:**
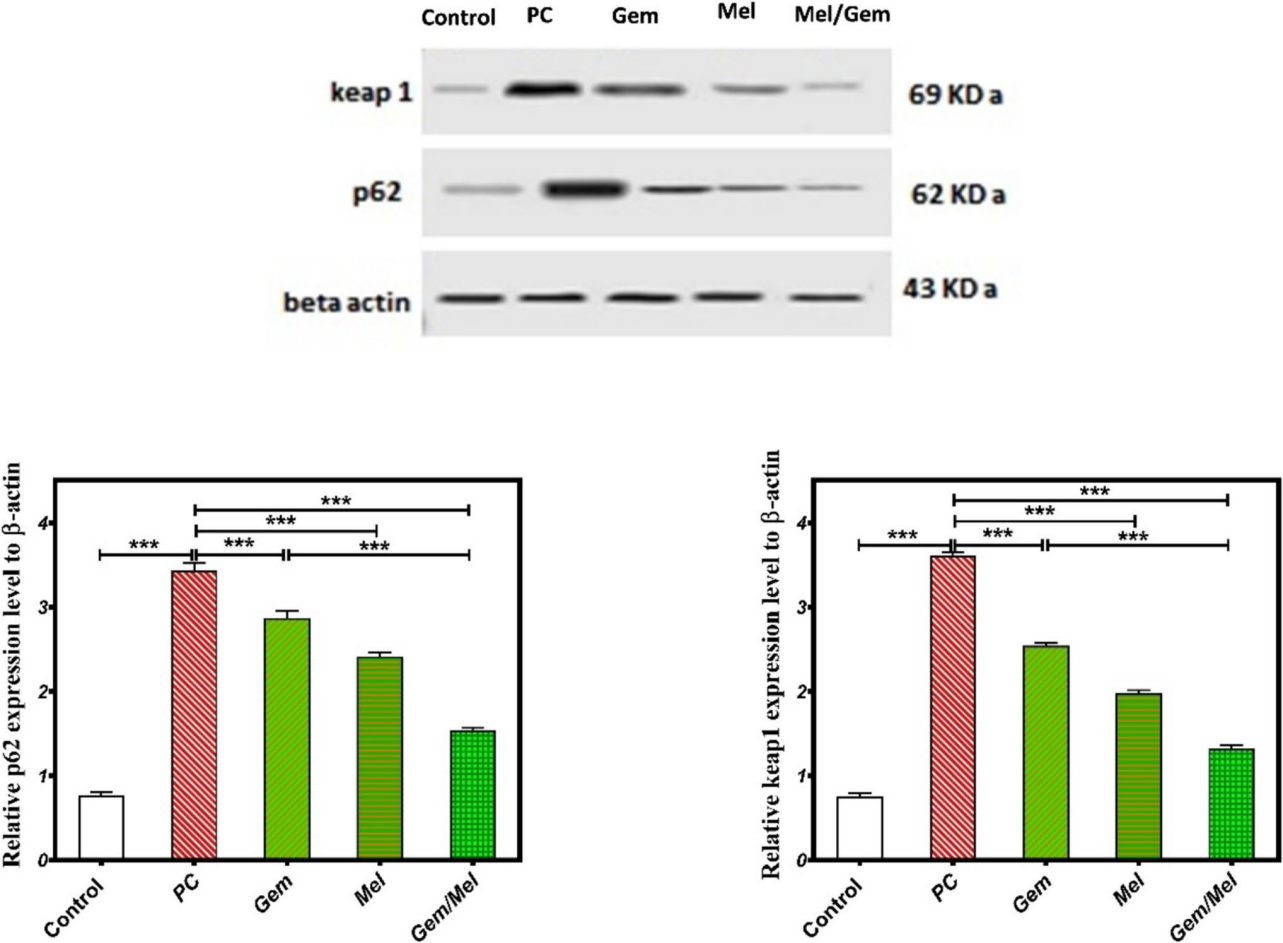
Melatonin/Gem influences the p62/keap1 signaling pathway. The effects of Mel on p62 and Keap1 protein expression were assessed among different groups utilizing the western blotting technique. Results are displayed as mean ± SEM, with statistical significance denoted by * relative to the control group (***P ≤ 0.0001)

The p62/Keap1 signaling pathway is known to be dysregulated in cancer. To investigate the molecular mechanism underlying Mel’s effect on pancreatic cancer, protein levels of p62 and Keap1 were measured in rats co-treated with Mel, Gem, or their combination. The PC group exhibited a significant elevation in p62 protein levels, with an increase of 243% (*P* ≤ 0.001) regarding the control group. Conversely, treatments with Mel, Gem, and the Mel/Gem combination resulted in reductions in p62 levels by 16.3%, 29.6%, and 55% (*P* ≤ 0.001), respectively, relating to the PC group. Notably, the combination of Mel and Gem resulted in a further reduction of p62 protein levels by 46.2% (*P* ≤ 0.001) in relation to the Gem treatment group alone (Fig. 4).

Similarly, the PC group indicated a marked rise in Keap1 protein expression, showing an increase of 377.7% (*P* ≤ 0.001) relating to the control group. Administration of Mel, Gem, and their combined treatment significantly diminished Keap1 levels by 29.6%, 45.2%, and 63.3% (*P* ≤ 0.001), respectively, corresponding to the PC group. Notably, the combined use of Mel and Gem resulted in an additional 47.9% reduction in Keap1 protein levels (*P* ≤ 0.001) concerning treatment with Gem alone (Fig. 4).

### Melatonin/Gem attenuates pancreatic fibrosis in rats

**Fig. 5:**
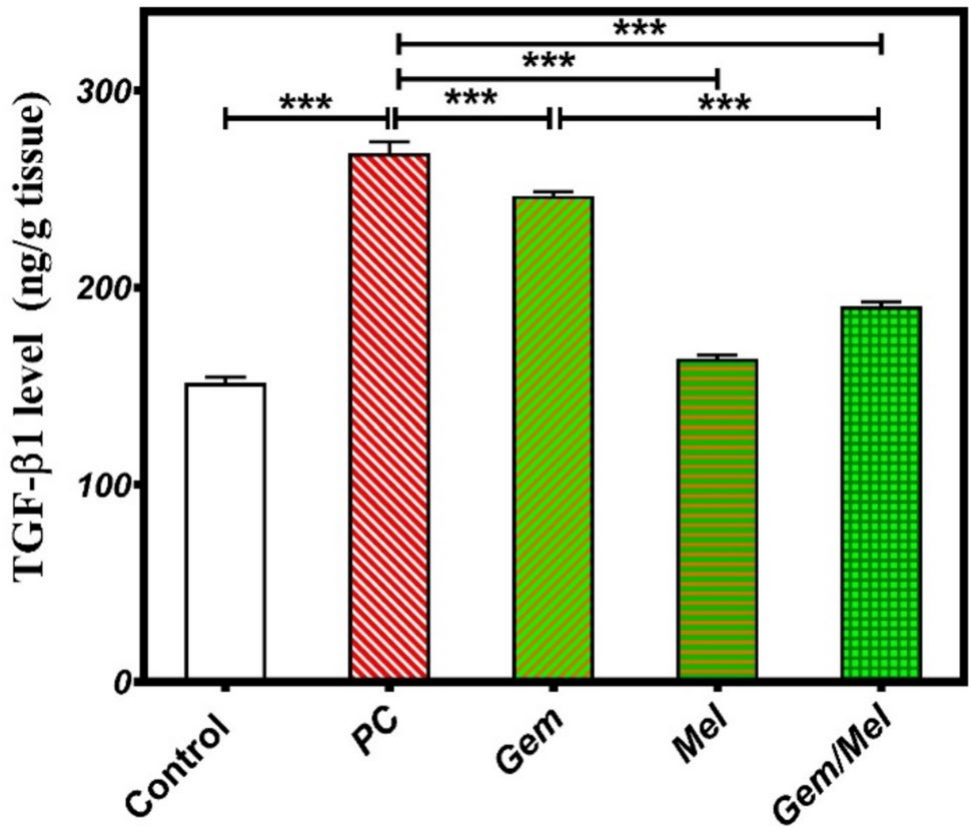
Melatonin enhances the antifibrotic effects of Gem in pancreatic cancer. Antifibrotic activity was evaluated in various groups by measuring TGF-β1 protein levels through the ELISA technique. Results are displayed as mean ± SEM, with statistical significance indicated by * relative to the control group (***P ≤ 0.0001)

The effects of Mel, either alone or combined with Gem, on TGF-β1 protein expression, a critical fibrotic marker, were investigated. In the PC group, TGF-β1 protein levels showed a notable increase of 77% (*P* ≤ 0.001) in relation to the control group. Treatments with Gem and Mel significantly decreased TGF-β1 levels by 8% and 38.9% (*P* ≤ 0.001), respectively, regarding the PC group. Moreover, rats treated with the Mel/Gem combination displayed significant reductions in TGF-β1 protein expression by 29% and 22.8% (*P* ≤ 0.001) concerning the PC and Gem groups, respectively (Fig. 5).

### Melatonin potentiates Gem antioxidant activities

**Fig. 6:**
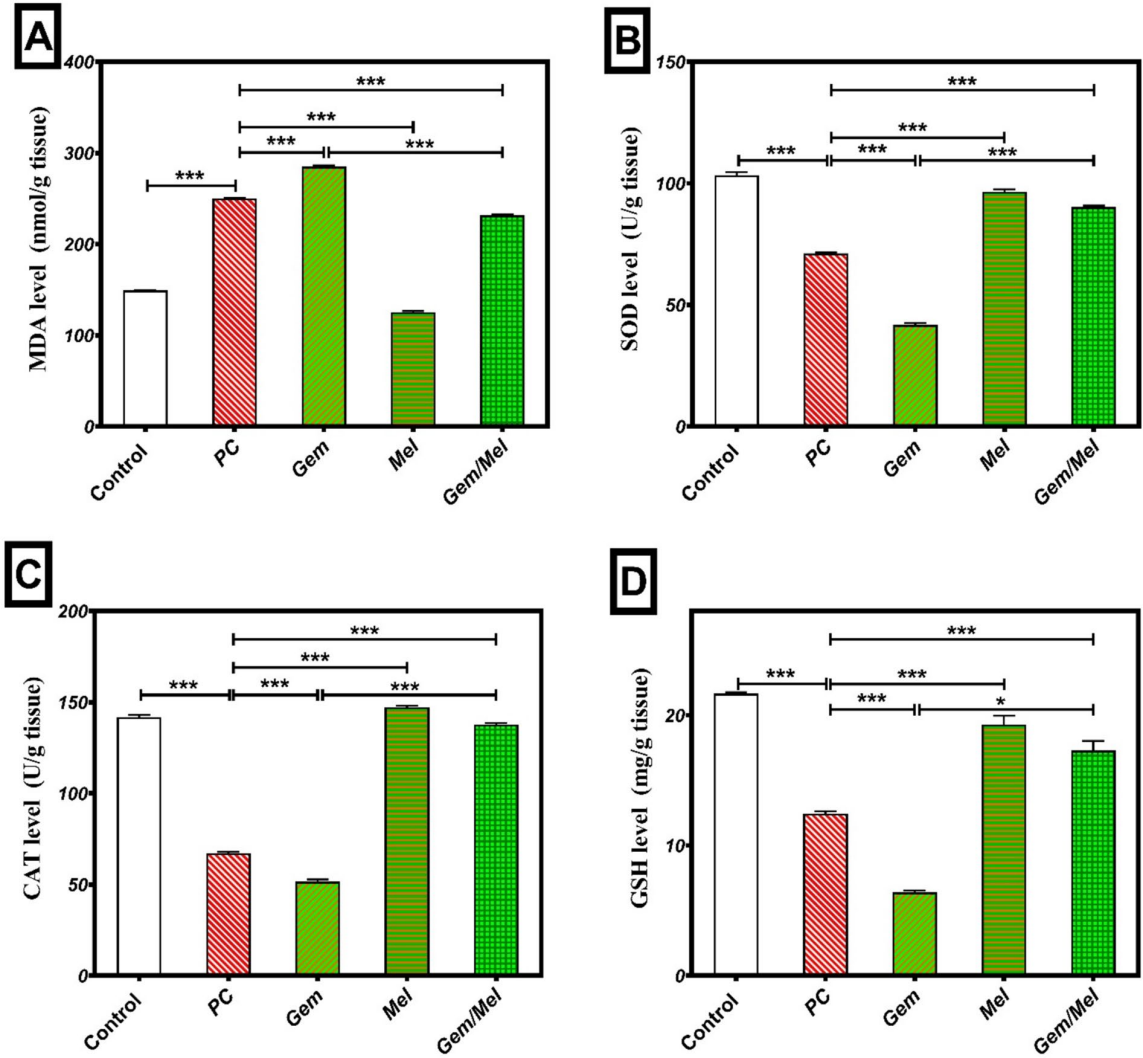
Melatonin enhances the antioxidant effects of Gem. Oxidative stress was assessed by quantifying pancreatic levels of MDA (**A**), SOD (**B**), CAT (**C**), and GSH (**D**). Results are expressed as mean ± SEM, with statistical significance denoted by ***P ≤ 0.001 and *P ≤ 0.05

The antioxidant potential of Mel was assessed by evaluating reactive oxygen species through the measurement of pancreatic levels of MDA, SOD, CAT, and GSH. In the PC group, pancreatic MDA levels were significantly elevated by 67% (*P* ≤ 0.001) as regards the control group. Treatment with Gem resulted in a 14% (*P* ≤ 0.001) elevate in MDA levels relating to the PC group. In contrast, Mel and Mel/Gem treatments significantly reduced pancreatic MDA levels by 49.8% and 7.3% (*P* ≤ 0.001), respectively, relating to the PC group. Furthermore, the Mel/Gem combination group demonstrated an 18.8% (*P* ≤ 0.001) reduction in MDA levels regarding the Gem-treated group (Fig. 6A).

Pancreatic SOD levels were significantly reduced in the PC and Gem groups, decreasing by 31.1% and 41.3% (*P* ≤ 0.001) regarding the control and PC groups, respectively. Conversely, the Mel and Mel/Gem treatment groups exhibited notable increases in pancreatic SOD levels, with rises of 35.6% and 26.7% (*P* ≤ 0.001), respectively, in relation to the PC group. Moreover, the Mel/Gem combination group showed a pronounced increase in pancreatic SOD levels, with an elevation of 115.9% (*P* ≤ 0.001) relating to the Gem group (Fig. 6B).

Pancreatic CAT levels in the PC group were significantly reduced by 52.6% (*P* < 0.001) relating to the control group. The Gem-treated group also showed a notable decrease in CAT levels, with a reduction of 23% (*P* ≤ 0.001) corresponding to the PC group. On the other hand, the Mel and Mel/Gem groups exhibited significant increases in pancreatic CAT levels, with rises of 118.9% and 105% (*P* ≤ 0.001), respectively, as regards the PC group. Remarkably, the Mel/Gem combination group demonstrated a 166.7% (*P* ≤ 0.001) increase in pancreatic CAT levels regarding the Gem group (Fig. 6C).

In the PC and Gem groups, pancreatic GSH levels were markedly decreased by 42.6% and 48.4% (*P* ≤ 0.001) relating to the control and PC groups, respectively. On the other hand, significant increases in pancreatic GSH levels were observed in the Mel and Mel/Gem groups, rising by 55% and 39.2% (*P* ≤ 0.001), respectively, corresponding to the PC group. Notably, the Mel/Gem combination group displayed a substantial enhancement in GSH levels, showing an increase of 169.9% (*P* ≤ 0.05) regarding the Gem group (Fig. 6D).

### Melatonin potentiates Gem anti-inflammatory activities

**Fig. 7:**
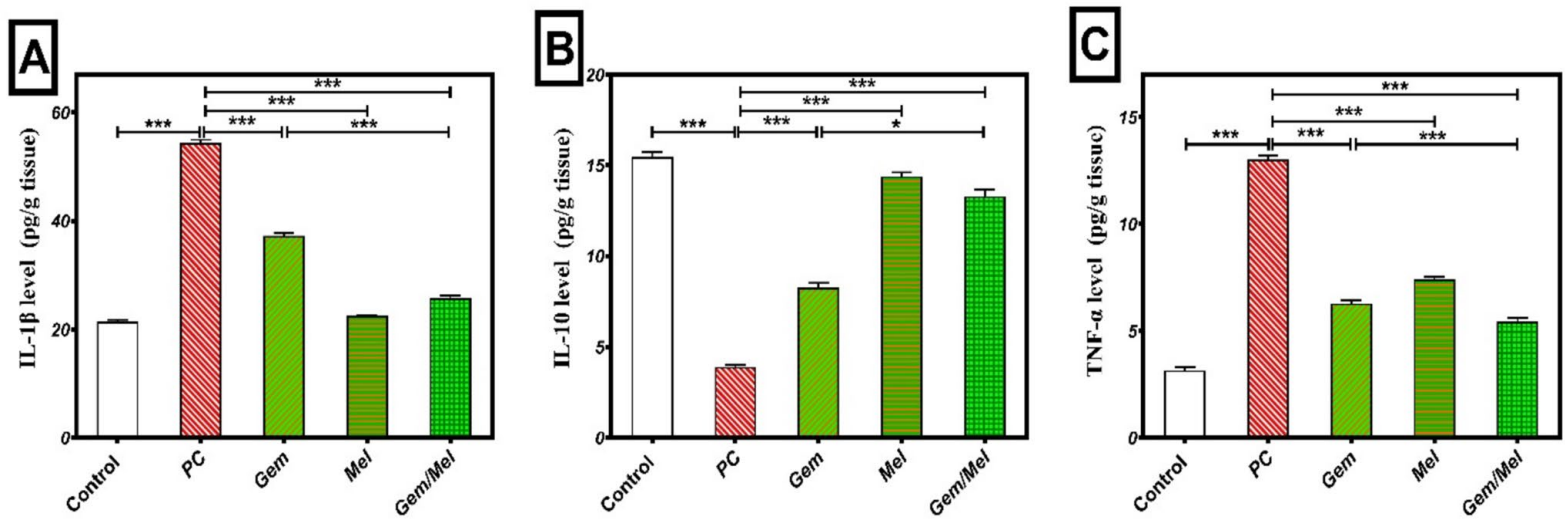
Melatonin enhances the anti-inflammatory effects of Gem. Anti-inflammatory activity was assessed by quantifying levels of IL-1β (**A**), IL-10 (**B**), and TNF-α (**C**) using the ELISA technique. Results are expressed as Mean ± SEM, with statistical significance denoted by ***P ≤ 0.001 and *P ≤ 0.05

The anti-inflammatory effects of Mel were assessed by measuring pancreatic levels of IL-1β, IL-10, and TNF-α. Pancreatic IL-1β levels in the PC group were significantly elevated by 153.6% (*P* ≤ 0.001) relating to the control group. Treatment with Gem, Mel, and their combination (Mel/Gem) significantly reduced pancreatic IL-1β levels by 31.6%, 58.6%, and 52.5% (*P* ≤ 0.001), respectively, as regards the PC group. In addition, the Mel/Gem combination group showed a 30.6% (*P* ≤ 0.001) further reduction in IL-1β levels relating to the Gem group (Fig. 7A).

Pancreatic IL-10 levels were significantly reduced in the PC group, showing a 74.7% decrease (*P* < 0.001) in relation to the control group. Conversely, treatments with Gem, Mel, and Mel/Gem resulted in significant increases in IL-10 levels by 111.9%, 268.4%, and 240.3% (*P* < 0.001), respectively, corresponding to the PC group. Moreover, the Mel/Gem combination group exhibited a substantial 60.6% (*P* < 0.05) increase in pancreatic IL-10 levels regarding the Gem group (Fig. 7B).

Pancreatic TNF-α levels in the PC group were significantly elevated, with a 311.6% increase (*P* < 0.001) as regards the control group. Treatment with Gem, Mel, and their combination (Mel/Gem) significantly decreased TNF-α levels by 51.6%, 43%, and 58% (*P* < 0.001), respectively, corresponding to the PC group. Additionally, the Mel/Gem combination group showed a further 13.5% (*P* < 0.001) reduction in TNF-α levels regarding the Gem group (Fig. 7C).

### Melatonin attenuates Gem-induced nephrotoxicity

**Fig. 8:**
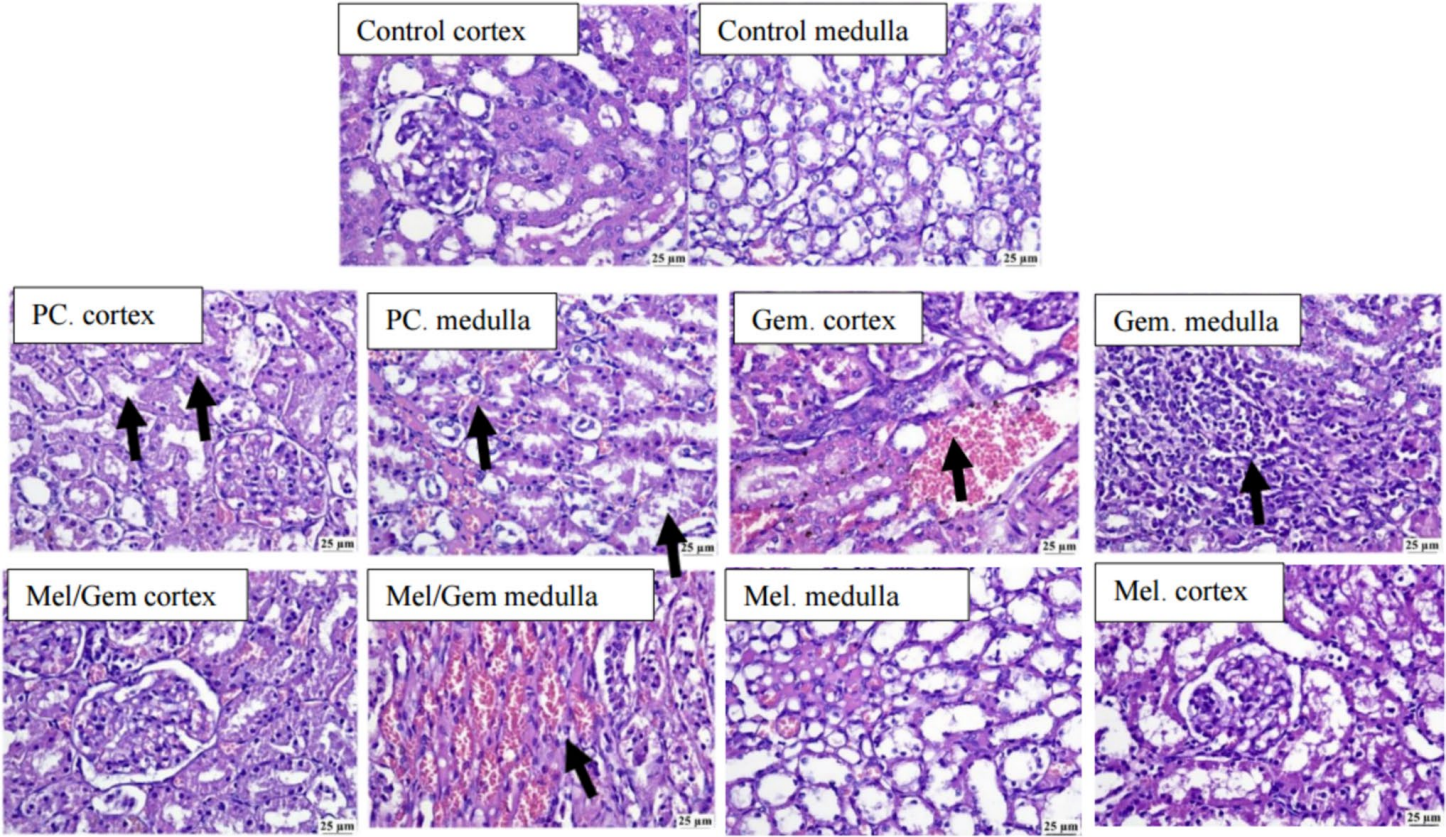
Melatonin alleviates Gem-induced nephrotoxicity. The protective effect of Mel against Gem-induced nephrotoxicity was evaluated in different groups through histopathological analysis of the renal cortex and medulla using H&E staining. Black arrows indicate areas of mononuclear inflammatory cell infiltration

H&E-stained kidney sections (Fig. 8) from the control group (A) displayed a normal histological structure of both the renal cortex and medulla. In contrast, the PC group (B) and Gem group (C) exhibited significant pathological changes, including marked vascular congestion and infiltration of perivascular mononuclear inflammatory cells, with pronounced mononuclear inflammatory cell infiltration in the renal medulla. Kidney sections from the Mel group (E) showed an apparently normal histological structure of the renal cortex and medulla. Notably, the Mel/Gem group (D) demonstrated considerable improvement, with kidney sections revealing only a few dilated blood vessels in the renal medulla, visible under 25 μm magnification.

## Discussion

In cancer treatment, the emergence of drug resistance remains a significant challenge, prompting intensive investigation into novel targeted therapeutic strategies. Gem, a well-established therapeutic agent for PC, is encountered by limitations such as resistance to treatment and side effects like nephrotoxicity. To overcome these challenges, targeting the P62/Keap-1 pathway has emerged as a potential strategy to mitigate cancer cell resistance to anticancer agents (Zhu et al. 2020).

The stress-inducible adaptor protein p62/SQSTM1 (commonly referred to as p62) functions as a multifunctional adapter in numerous signaling pathways, especially in autophagy, where its intracellular levels act as a marker of autophagic flux (Sánchez-Martín and Komatsu 2018). As a secondary autophagy marker, p62 interacts directly with microtubule-related protein 1 light chain 3 II (LC3II) within autophagosomes and is degraded during autophagy progression. A reduction in p62 levels signifies the completion of autophagic processes (Ma et al. 2018), whereas its accumulation is indicative of autophagy deficiency (Huang et al. 2020). p62 plays a pivotal role in PC development and has been identified through immunohistochemical staining in the cytoplasm of pancreatic carcinoma cells (Zhang et al. 2020). This association highlights the potential link between p62 accumulation and cancer progression.

Elevated p62 expression was found as an indicator for pancreatic carcinoma and has been correlated with poorer survival outcomes (Zhang et al. 2020). These findings underscore that p62 expression increases substantially with the development of PC; therefore, P62 may be new targets for future carcinoma therapy. Treatment with Mel or Gem alone resulted in reduced p62 expression, aligning with findings by Yan et al. (2020) and Marchand et al. (2023), respectively (Yan et al. 2020, Marchand et al. 2023). Notably, the combination of Mel and Gem was more effective than either agent alone in reducing p62 expression and exerting anticancer effects. Prior studies have supported the role of autophagy induction in enhancing the anti-tumor efficacy of Gem, as shown in the pancreatic cancer cell line MIA PaCa-2 (Kim et al. 2017). Additionally, Fernandez-Gil et al. (2019) and Leja-Szpak et al. (2018) demonstrated that Mel enhances the cytotoxic effects of cisplatin and Gem in neck and head squamous cell carcinoma and pancreatic carcinoma cells, respectively (Leja-Szpak et al. 2018, Fernandez-Gil et al. 2019). Therefore, the enhanced anti-tumor activity observed with the combination of Mel and Gem may be attributed to Mel’s ability to downregulate p62 expression.

A prominent feature of PC is its unique tumor microenvironment (TME), which is marked by an intense desmoplastic stromal reaction resulting in a collagen-dense extracellular matrix (ECM) (Ho et al. 2020). The TME in PC is particularly rich in transforming growth factor β (TGF-β), a critical immunosuppressive cytokine that plays a pivotal role in PC progression and the evasion of immune responses (Luo et al. 2023; Yang et al. 2010). TGF-β1 is fundamental for both the development and persistence of the cancer-associated fibroblasts (CAFs) phenotype (Shi et al. 2020). Furthermore, a connection has been identified between P62 and TGF-β, as TGF-β signaling can enhance tumor progression by promoting macroautophagy (Trelford and Di Guglielmo 2020). In contrast, research by Kim et al. (2019) demonstrated that autophagy inhibition counteracted the antifibrotic effects of IL-37 in lung cancer, highlighting autophagy’s significant contribution to IL-37’s antifibrotic impact in bleomycin-induced lung damage (Kim et al. 2019). Similarly, Kang et al. (2021) reported that p62 deficiency hindered CAF activation, reduced TGF-β1 production, and consequently inhibited tumor growth (Kang et al. 2021). In our findings, the PC xenograft model exhibited elevated TGF-β1 protein expression, which was effectively suppressed by treatment with Mel and Gem. Supporting these results, previous studies have established that elevated TGF-β levels in PC (Jian et al. 2023). Additionally, TGF-β induces oxidative stress by both increasing the production of ROS and weakening antioxidant defenses, resulting in DNA damage and promoting the survival of cancer cells (Chung et al. 2021).

Enhanced levels of ROS accelerate the rate of genetic mutations, thereby intensifying oncogene activation and contributing to cancer progression (El-Gamil et al. 2024, Shaldam et al. 2024). The nuclear factor erythroid 2-related factor 2 (Nrf2)/Keap-1 pathway plays a pivotal role in regulating antioxidant protein expression, where Nrf2 associates with Keap-1, which keeps Nrf2 in an inactive state under normal physiological conditions (Ulasov et al. 2022). Research by Jiang et al. (2015) demonstrated that following autophagy activation and increased autophagy flux, Keap1-p62 complexes are sequestered into autophagosomes, leading to Keap-1 degradation via lysosomal pathways and the modulation of Nrf2 activity. This transient activation mechanism of Nrf2 mirrors the protective effects of its canonical activation. Moreover, Nrf2 facilitates the antioxidant response element (ARE)-driven expression of p62, highlighting the interconnected roles of autophagy and Nrf2 in restoring cellular homeostasis (Jiang et al. 2015).

Our findings revealed that Mel significantly reduced Keap-1 expression and MDA levels while simultaneously prompting the activity of key antioxidant markers, including SOD, GSH, and CAT. Melatonin’s antioxidant activity is mediated through both direct and indirect mechanisms. Directly, Mel neutralizes oxidants via its non-receptor-mediated free radical scavenging properties. Indirectly, it mitigates oxidative stress by activating antioxidant enzymes, inhibiting pro-oxidative enzymes, and stabilizing the mitochondrial inner membrane, thereby preserving mitochondrial integrity, reducing electron leakage, and minimizing ROS production (Madhu et al. 2021). Previous research has shown that Mel suppresses p62 expression in colitis-associated colon carcinogenesis in mice, an effect correlated with increased Nrf2 levels and the upregulation of its downstream antioxidant enzymes (Trivedi et al. 2016). Consequently, the activation of the Nrf2-Keap1 pathway by Mel is likely associated with autophagy regulation through p62.

Inflammation associated with tumors, a defining feature of cancer, plays a significant role in tumorigenesis and progression by supplying bioactive molecules within the TME (Hefny et al. 2024a, Tawfik et al. 2024). This inflammatory process facilitates nascent neoplasms in acquiring additional hallmark traits (Hibino et al. 2021, Hefny et al. 2024b). The protein p62 is implicated in various cellular functions through its interactions with multiple intracellular signaling pathways, including NF-κB (Ning and Wang 2019, Hennig et al. 2021). Moreover, TGF-β enhances a pro-inflammatory TME by activating inflammatory cytokines, interacting with NF-κB, and recruiting immune cells (Chung et al. 2021). NF-κB has long been recognized as a potential mediator linking inflammation to cancer (Staal and Beyaert 2018). Elevated NF-κB activity can induce the expression of numerous pro-survival genes, contributing to resistance against chemotherapy (Pavitra et al. 2023). Experimental evidence from mouse models has demonstrated that the accumulation of p62 stimulates NF-κB signaling, thereby accelerating pancreatic cancer development (Qian et al. 2020).

The migration of activated NF-κB dimers to the nucleus stimulates the transcription of genes associated with inflammation, including IL-1β (Antar et al. 2024, El-Dessouki et al. 2024a). IL-1β amplifies the inflammatory response by sustaining NF-κB activation, promoting the production of additional pro-inflammatory cytokines, and enhancing macrophage recruitment (El-Dessouki et al. 2024b). Additionally, IL-1β is pivotal in facilitating cancer progression by promoting metastasis, angiogenesis, and tumor development. Evidence presented by Bent et al. (2018) indicates that elevated IL-1β levels are linked to the suppression of adaptive immunity, as well as the facilitation of tumor progression and metastasis (Bent et al. 2018). Similarly, IL-10, an immune-regulatory cytokine produced by macrophages, T lymphocytes, and NK cells, exhibits immunosuppressive and anti-angiogenic properties. IL-10 counteracts tumorigenesis by downregulating (vascular endothelial growth factor) VEGF, IL-1β, TNF-α, IL-6, and matrix metalloproteinase-9 (MMP-9) while inhibiting NF-κB signaling (Sheikhpour et al. 2018). However, studies suggest that reduced IL-10 expression permits increased angiogenesis and heightened activity of VEGF, IL-1β, TNF-α, IL-6, and NF-κB, ultimately enhancing tumor growth. Research by Aziz et al. (2022) demonstrated that IL-10 suppression accelerates tumor development, aligning with our observations (Aziz et al. 2022). In our study, the induction of PC was linked to a significant upregulation of inflammatory markers such as TNF-α and IL-1β, alongside a marked reduction in IL-10 expression.

Although Gem demonstrates significant therapeutic benefits, its administration is associated with a 2.9% incidence of acute kidney injury (AKI) (Lyrio et al. 2024). Histological examination of renal tissues in this context revealed multiple pathological alterations, including pronounced degeneration and necrosis of renal tubules, thickened vascular walls with thrombus formation, infiltration of mononuclear inflammatory cells, cystic changes in the interstitial tissue, glomerular lobulation, and expansion of Bowman’s space accompanied by hyaline casts within renal tubules. These findings align with the study by Mortazavi et al. (2017), which reported severe renal damage in gemcitabine-treated rats, characterized by tubular degeneration, necrosis, and inflammatory cell infiltration within the kidney interstitium. Gemcitabine thus induces notable adverse effects, with renal damage manifesting as vascular congestion, inflammation, necrosis, and significant infiltration of inflammatory cells (Mortazavi et al. 2017).

Additionally, Gem-induced thrombotic microangiopathy is a well-documented complication, characterized by a spectrum of clinical manifestations such as thrombocytopenia, microangiopathic hemolytic anemia, and varying levels of neurological and renal dysfunction (Katagiri and Hinoshita 2018, Madkhali 2021). To address these adverse effects while maintaining the therapeutic efficacy of Gem, our study employed a combination therapy with Mel. Consistent with prior research, Mel demonstrated a renal protective effect against Gem-induced nephrotoxicity. Previous findings indicated that Mel administration counteracted reduced antioxidant levels caused by cadmium (Cd) exposure, protecting renal cells from Cd-induced oxidative damage through free radical scavenging and enhancing cellular antioxidant defense (Yang et al. 2023). Accordingly, Mel may mitigate Gem-induced nephrotoxicity by reducing oxidative stress and modulating inflammatory pathways.

## Conclusion

**Fig. 9:**
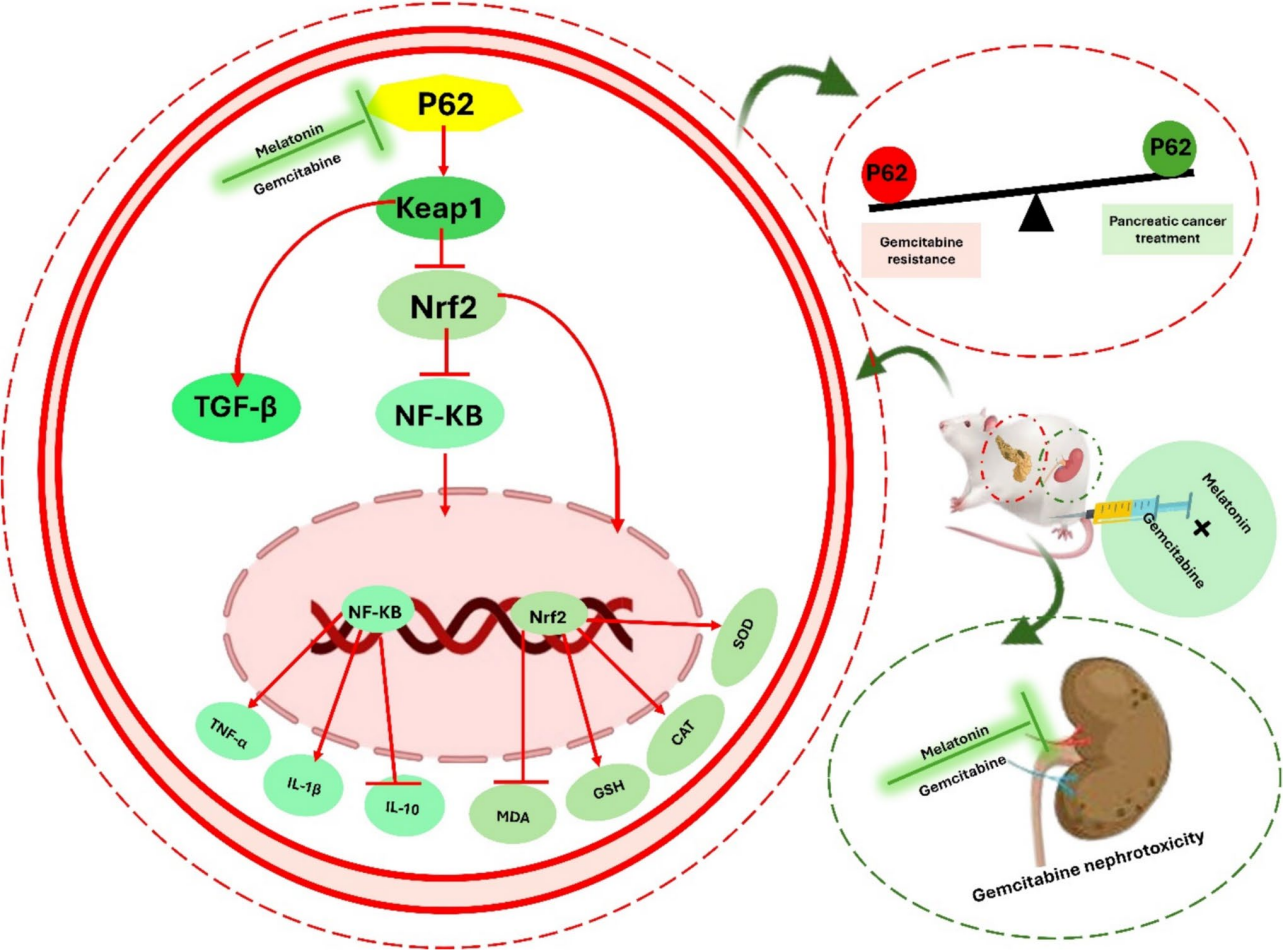
Proposed mechanism of melatonin action. Melatonin enhances the anti-tumor efficacy of gemcitabine while reducing its nephrotoxic effects in pancreatic cancer by inhibiting the P62/Keap1 pathway. This inhibition suppresses the transcription and translation of downstream genes that play critical roles in promoting fibrosis, oxidative stress, and inflammation, thereby addressing key pathological processes associated with pancreatic cancer

Melatonin therapy demonstrated significant protective effects on renal tissues compromised by Gem treatment, notably improving outcomes by targeting inflammation and oxidative stress through the modulation of the P62/Keap1 pathway. The combined administration of Mel and Gem resulted in a pronounced downregulation of the P62/Keap1 pathway compared to the use of either agent alone. These findings suggest a synergistic interaction between Mel and Gem in mitigating renal toxicity as illustrated in Fig. 9. Future studies should explore the precise molecular interactions between melatonin and autophagy regulators to fully elucidate its role in pancreatic cancer therapy and explore the specific role of P62-mediated autophagy in the context of PC to better understand its therapeutic implications.

## Data Availability

All data and materials can be provided upon a reasonable request. All source data for this work (or generated in this study) are available upon reasonable request.
